# Tripartite Motif Containing 11 Interacts with DUSP6 to Promote the Growth of Human Osteosarcoma Cells through Regulating ERK1/2 Pathway

**DOI:** 10.1155/2019/9612125

**Published:** 2019-12-25

**Authors:** Zhaofeng Wang, Xiaobo Xu, Wenxiao Tang, Youcai Zhu, Jichao Hu, Xingen Zhang

**Affiliations:** ^1^Department of Clinical Laboratory, Zhejiang Rongjun Hospital, Jiaxing, Zhejiang, China; ^2^Department of Chest Disease Center, Zhejiang Rongjun Hospital, Jiaxing, Zhejiang, China; ^3^Department of Orthopedics, Zhejiang Rongjun Hospital, Jiaxing, Zhejiang, China

## Abstract

Tripartite Motif Containing 11 (TRIM11), an E3 ubiquitin ligase, is identified as a carcinogen causing certain human cancers. However, the specific role of TRIM11 is still uncovered in human osteosarcoma (OS) cells. To explore the role of TRIM11 in OS cells, TRIM11 was induced by silencing and overexpression in OS cells using RNA interference (RNAi) and lentiviral vector, respectively. qRT-PCR and western blot were used to examine the transcription and translation levels of the target gene. Cell count kit-8 (CCK-8) assays were established to analyze cell proliferation. Cell apoptosis ratio was determined via flow cytometry. In our analyses, TRIM11 was suggested to be upregulated, and it functioned as a pro-proliferation and antiapoptosis factor in OS cells. Moreover, the extracellular-signal-regulated kinase 1/2 (ERK1/2) inhibitor PD98059 was used to examine the relationship between TRIM11 and ERK1/2 in OS cells. Results demonstrated that the role of TRIM11 was significantly disrupted by the ERK1/2 inhibitor PD98059. Interestingly, we found TRIM11 overexpression did not affect dual-specificity phosphatase 6 (DUSP6) transcription, but improved its translation in OS cells. Co-immunoprecipitation (Co-IP) analyses revealed that TRIM11 interacted with DUSP6. Importantly, overexpression of TRIM11 enhanced DUSP6 ubiquitination in OS cells. Therefore, TRIM11 might suppress the translation of DUSP6 via improving its ubiquitination. Additionally, TRIM11 silencing in OS cells significantly reduced its tumorigenicity *in vivo.* Overall, our findings firstly revealed that TRIM11 was an oncogene gene in the growth of OS cells and illustrated its potential function as a target in the treatment of OS.

## 1. Introduction

Osteosarcoma (OS) is a most frequent primary malignancy of bone, which is identified by the presence of malignant mesenchymal cells [1]. Frequently, OS mainly arises in children and adolescents [2]. Due to the early metastatic potential and highly aggressive nature of OS, the outcome of surgical resection and chemotherapy is far from being satisfactory [3]. Although tumor diagnosis and treatments have been greatly improved in recently years, the prognosis of patients with recurrence and metastasis remains poor [4]. Therefore, the effective biomarkers for OS early diagnosis and prognosis are urgently desired [5]. However, the molecular mechanism of OS is not fully explored.

Tripartite Motif Containing 11 (TRIM11) belongs to the TRIM protein family, which is defined as an E3 ubiquitin ligase containing a coiled-coil region, a PRY domain, and a SPRY domain [6]. Previous reports have indicated that TRIM11 overexpression promotes the progression of lung cancer and gliomas cells [7, 8]. Moreover, TRIM11 is a direct target of miR-24-3p in colon cancer cells [9]. Furthermore, downregulation of TRIM11 has contributed to the treatment of breast cancer cells through inhibiting ERK1/2 and JNK1/2 signaling pathways [10]. However, the detailed function of TRIM11 is still uncovered in OS cells.

Dual-specificity phosphatase 6 (DUSP6) is a cytosolic phosphatase, which is a negative feedback regulator for the extracellular-signal-regulated kinase 1/2 (ERK1/2) [11]. It has been confirmed that DUSP6 has a neuroprotective effect on A*β*-induced cytotoxicity via suppressing ERK1/2 activation in neural stem cells [12]. Moreover, DUSP6 downregulation has led to the progression and differentiation of esophageal squamous cell carcinoma (ESCC) [13]. Furthermore, a growing body of evidence has demonstrated that DUSP6 is a tumor suppressor in lung cancer cells [14, 15]. Importantly, TRIM7 has improved the proliferation of hepatocellular carcinoma cells through the DUSP6/p38 pathway [16]. Knockdown of TRIM11 significantly inhibited the transcription of DUSP6 in D-54 glioblastoma multiforme (GBM) cells [8]. However, the precise connection between TRIM11 and DUSP6 is less explored in OS cells.

In the present research, we aimed to investigate the role of TRIM11 in OS cells. TRIM11 was induced via silencing and overexpression by using RNA interference (RNAi) and lentiviral vector in OS cells. Our findings not only illustrated the function of TRIM11 but also indicated its potential signaling pathway in OS cells.

## 2. Results

### 2.1. TRIM11 Was Upregulated in OS Cells

**Figure 1:**
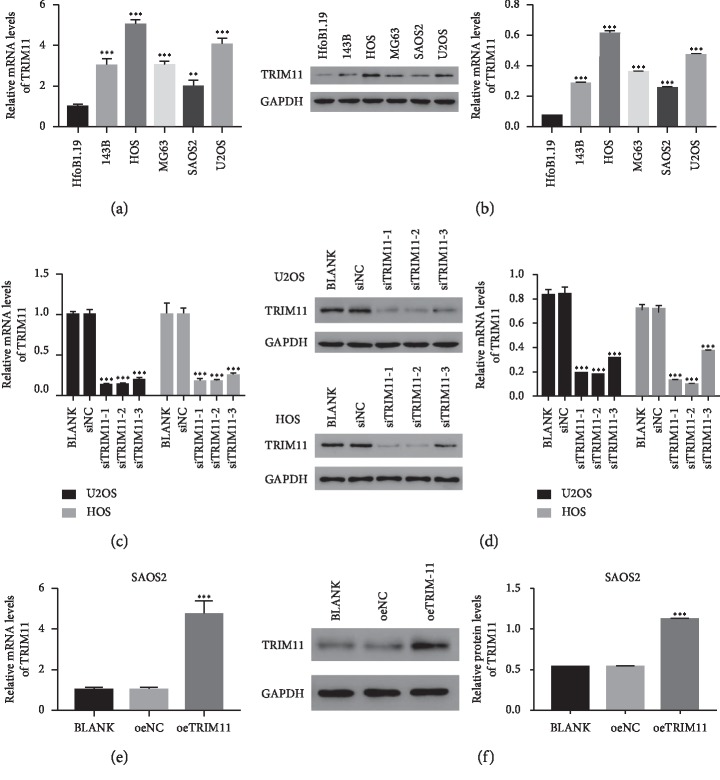
TRIM11 silencing and overexpression in OS cells. (a, b) TRIM11 was significantly upregulated in OS cells (143B, HOS, MG63, SAOS2, and U2OS) compared with that of HfoB1.19 cells. ^*∗∗*^*p* < 0.01 vs HfoB1.19 cells; ^*∗∗∗*^*p* < 0.001 vs HfoB1.19 cells. (c, d) The level of TRIM11 was significantly downregulated in siTRIM11 transfected cells. ^*∗∗∗*^*p* < 0.001 vs siNC. (e, f) The level of TRIM11 was upregulated in oeTRIM11 transfected cells. ^*∗∗∗*^*p* < 0.001 vs oeNC.

Firstly, we quantified the levels of TRIM11 in human normal hFOb1.19 osteogenesis cells and five OS cell lines, including 143B, HOS, MG63, SAOS2, and U2OS. Clearly, the relative mRNA and protein levels of TRIM11 were significantly improved in OS cells compared with those of hFOb1.19 cells, especially in HOS and U2OS cells (Figures 1(a) and 1(b)). These findings suggested that TRIM11 was upregulated in OS cells.

### 2.2. Silencing and Overexpression of TRIM11 in OS Cells

To address the function of TRIM11 in OS cells, we induced knockdown and overexpression of TRIM11 in OS cells. For TRIM11 silencing, three short interference RNAs (siRNAs; siTRIM11-1, siTRIM11-2, and siTRIM11-3) that target different regions of the human gene TRIM11 (NM_145214.2) were synthesized. Then, all the TRIM11 siTRIM11s were transfected into U2OS and HOS cells, respectively. Meanwhile, a nonspecific scrambled siRNA was functioned as negative control (siNC). Clearly, the level of TRIM11 was significantly downregulated in siTRIM11 transfected cells compared with that of siNC transfected cells in two cell lines. These results indicated that siTRIM11s worked well to silence the endogenous expression of TRIM11. Moreover, siTRIM11-1 and siTRIM11-2 presented a stronger effect than siTRIM11-3 in two cell lines (Figures 1(c) and 1(d)). Therefore, the following analyses were performed in siTRIM11-1 and siTRIM11-2 transfected cells.

Furthermore, the full length of TRIM11 cDNA was inserted into the lentiviral vector (pLVX-Puro). Then, the recombinant (oeTRIM11) vector and a mock (oeNC) vector were transfected into SAOS2 cells respectively. Obviously, the level of TRIM11 was obviously promoted in oeTRIM11 transfected cells than in oeNC transfected cells (Figures 1(e) and 1(f)).

### 2.3. TRIM11 Silencing Inhibited the Growth of OS Cells

**Figure 2:**
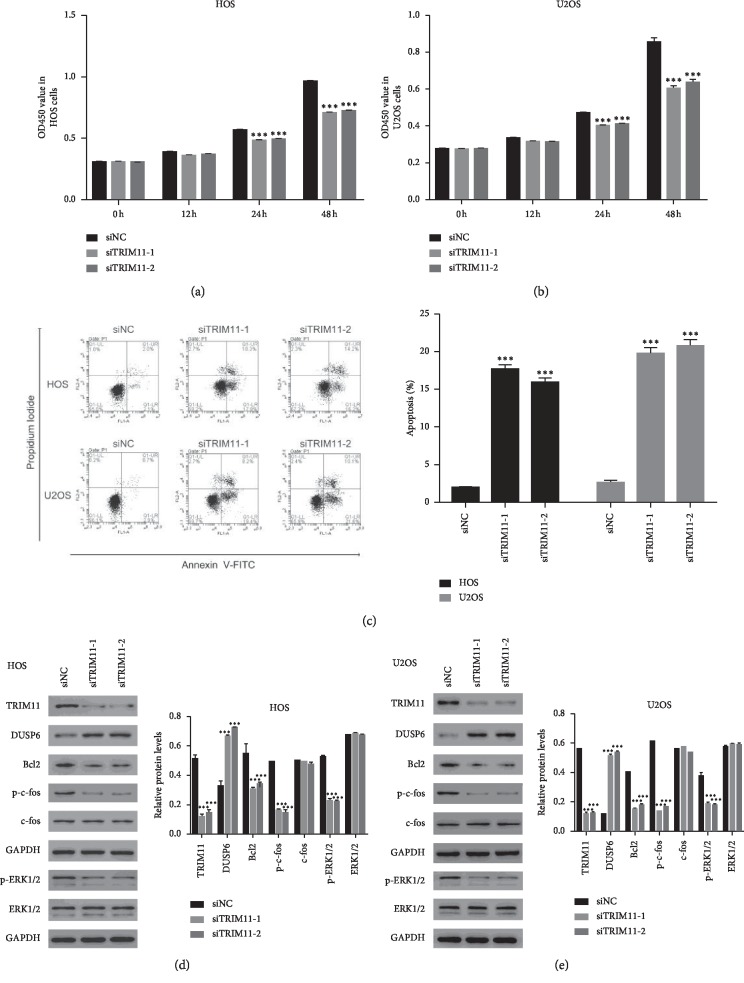
TRIM11 silencing suppressed the growth of OS cells. (a, b) Cell proliferation was determined in HOS and U2OS at 0 h, 12 h, 24 h, and 48 h after transfecting with siNC, siTRIM11-1, or siTRIM11-2. ^*∗∗∗*^*p* < 0.001 vs siNC. (c) Knockdown of TRIM11 promoted the apoptosis of HOS and U2OS. ^*∗∗∗*^*p* < 0.001 vs siNC. (d, e) Western blot was used to examine the protein content of TRIM11, DUSP6, Bcl2, c-fos, p-c-fos, p-ERK1/2, and ERK1/2 in HOS and U2OS cells as indicated above. ^*∗∗∗*^*p* < 0.001 vs siNC.

To further analyze the role of TRIM11 in OS cells, CCK-8 assays were used to determine the proliferation rate. Importantly, the cell proliferation rate was deeply decreased in HOS and U2OS cells that were transfected with siTRIM11-1 or siTRIM11-2 compared with that in siNC transfected cells (Figures 2(a) and 2(b)). Importantly, cell apoptosis analyses suggested that knockdown of TRIM11 remarkably improved the apoptosis of HOS and U2OS cells (Figure 2(c)). Overall, these results demonstrated that knockdown of TRIM11 inhibited the growth of OS cells.

Bcl2 is widely described as a representative of antiapoptosis proteins [17]. In this study, we found the level of Bcl2 was deeply downregulated in siTRIM11 transfected cells. These findings further demonstrated the antiapoptosis function of TRIM11 in OS cells. Moreover, knockdown of TRIM11 significantly improved the protein content of DUSP6 in HOS and U2OS cells. Interestingly, the phosphorylation of ERK1/2 was much lower in siTRIM11 transfected cells (Figures 2(d) and 2(e)). Furthermore, c-fos, a downstream target of ERK1/2, is the primary subunits of transcription factor activator protein-1 [18, 19]. Importantly, the phosphorylation of c-fos (p-c-fos) was also deeply suppressed in siTRIM11 transfected cells. Thus, these results suggested that TRIM11 might involve in the ERK1/2/c-fos pathway in OS cells.

### 2.4. The ERK1/2 Inhibitor PD98059 Suppressed the Function of TRIM11 in OS Cells

**Figure 3:**
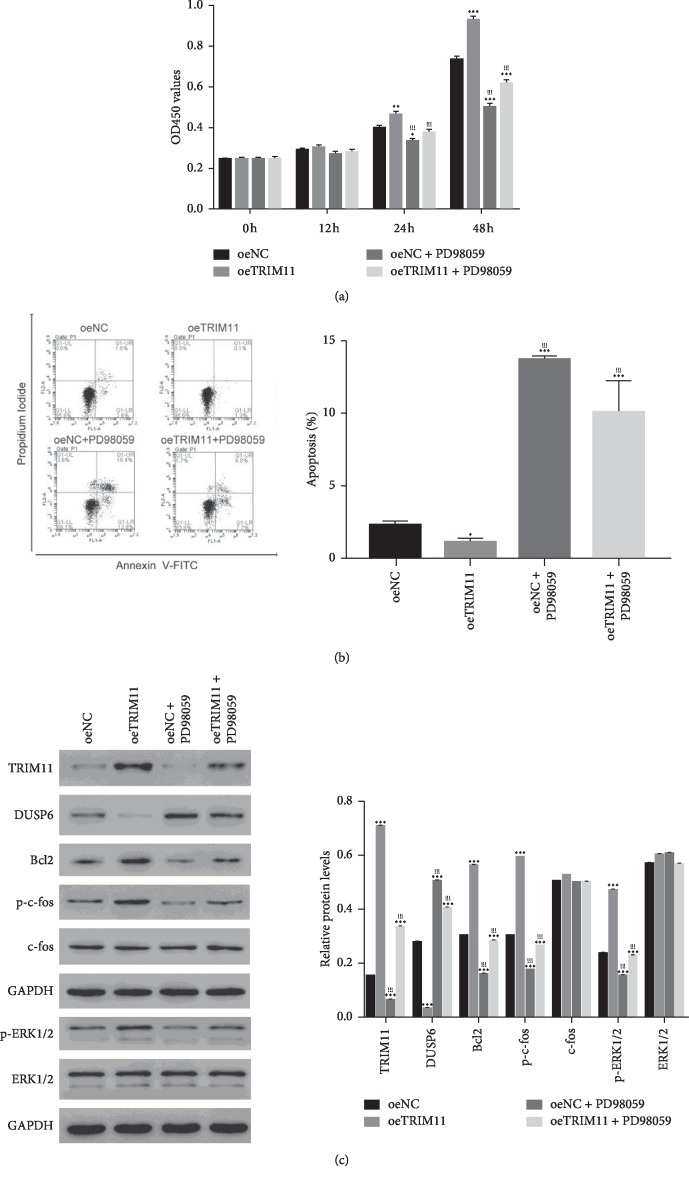
The function of TRIM11 was disrupted by the ERK1/2 inhibitor PD98059 in OS cells. (a) The ERK1/2 inhibitor PD98059 suppressed the proliferation rate of oeTRIM11 transfected cells. ^*∗*^*p* < 0.05 vs oeNC; ^*∗∗*^*p* < 0.01 vs oeNC; ^*∗∗∗*^*p* < 0.001 vs oeNC; ^!!!^*p* < 0.001 vs oeTRIM11. (b) The ERK1/2 inhibitor PD98059 significantly promoted the apoptosis ratio of oeTRIM11 transfected cells. ^*∗*^*p* < 0.05 vs oeNC, ^*∗∗∗*^*p* < 0.001 vs oeNC; ^!!!^*p* < 0.001 vs oeTRIM11. (c) Western blot was performed to determine the protein content of TRIM11, DUSP6, Bcl2, c-fos, p-c-fos, p-ERK1/2, and ERK1/2 in different cells as indicated above. ^*∗∗∗*^*p* < 0.001 vs oeNC; ^!!!^*p* < 0.001 vs oeTRIM11.

To examine the connection between TRIM11 and ERK1/2 in OS cells, the oeTRIM11 transfected cells were cultured in the presence of the ERK1/2 inhibitor PD98059. As presented in Figure 3(a), the proliferation rate of oeTRIM11 transfected cells was much higher than that of oeNC transfected cells. Nevertheless, TRIM11 pro-proliferation effect was blocked in the presence of the ERK1/2 inhibitor PD98059 (Figure 3(a)). Moreover, TRIM11 overexpression deeply suppressed the apoptosis of OS cells, whereas this suppression was released by the ERK1/2 inhibitor PD98059 (Figure 3(b)). Therefore, TRIM11 overexpression promoted the growth of OS cells, but it was disrupted by the ERK1/2 inhibitor PD98059.

Furthermore, overexpression of TRIM11 inhibited the expression of DUSP14, whereas promoted the levels of Bcl2 and p-c-fos. Much importantly, the phosphorylation of ERK1/2 was promoted in oeTRIM11 transfected cells, but deeply blocked in the presence of the ERK1/2 inhibitor PD98059 in OS cells (Figure 3(c)). Overall, all these findings illustrated that TRIM11 was negatively correlated with DUSP6. Much importantly, TRIM11 might promote the growth of OS cells through promoting the phosphorylation of ERK1/2.

### 2.5. TRIM11 Interacted with DUSP6 and Enhanced Its Ubiquitination in OS Cells

**Figure 4:**
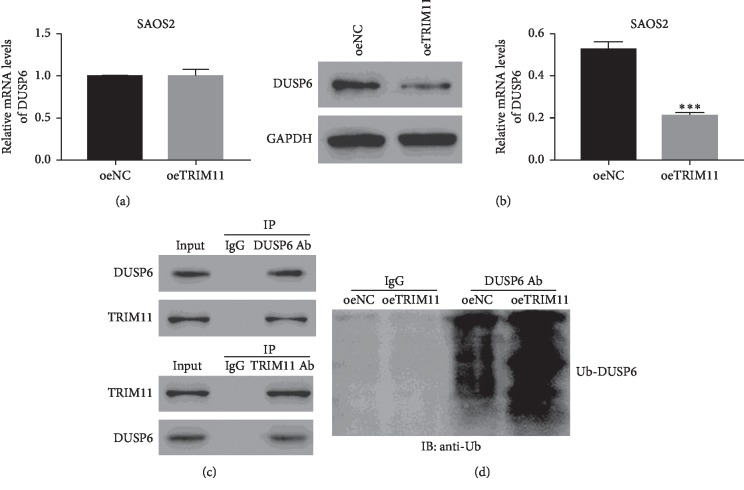
TRIM11 interacted with DUSP6 and enhanced its ubiquitination. (a, b) The relative mRNA and protein levels of DUSP6 were examined in SAOS2 cells that were transfected with oeNC or oeTRIM11. ^*∗∗∗*^*p* < 0.001 vs oeNC. (c) TRIM11 interacted with DUSP6 in OS cells. (d) Overexpression of TRIM11 enhanced the ubiquitination of DUSP6 in OS cells.

Next, we further examined the mRNA expression levels of DUSP6 in oeNC and oeTRIM11 transfected cells. Interestingly, the relative mRNA levels of DUSP6 showed no significant difference between oeNC and oeDUSP6 transfected cells, while the relative protein levels of DUSP6 was deeply downregulated in oeTRIM11 transfected cells (Figures 4(a) and 4(b)). Therefore, these results demonstrated that TRIM11 overexpression did not affect DUSP6 transcription, but deeply suppressed its translation in OS cells. Much importantly, the Co-IP assay confirmed that TRIM11 interacted with DUSP6 in OS cells (Figure 4(c)). Furthermore, we also examined DUSP6 ubiquitination in oeNC and oeTRIM11 transfected cells. Clearly, TRIM11 overexpression significantly enhanced the ubiquitination of DUSP6 in OS cells. Taken together, TRIM11 might inhibit the translations of DUSP6 through enhancing its ubiquitination in OS cells (Figure 4(d)).

### 2.6. Knockdown of TRIM11 Suppressed the Tumorigenicity of OS Cells *In Vivo*

**Figure 5:**
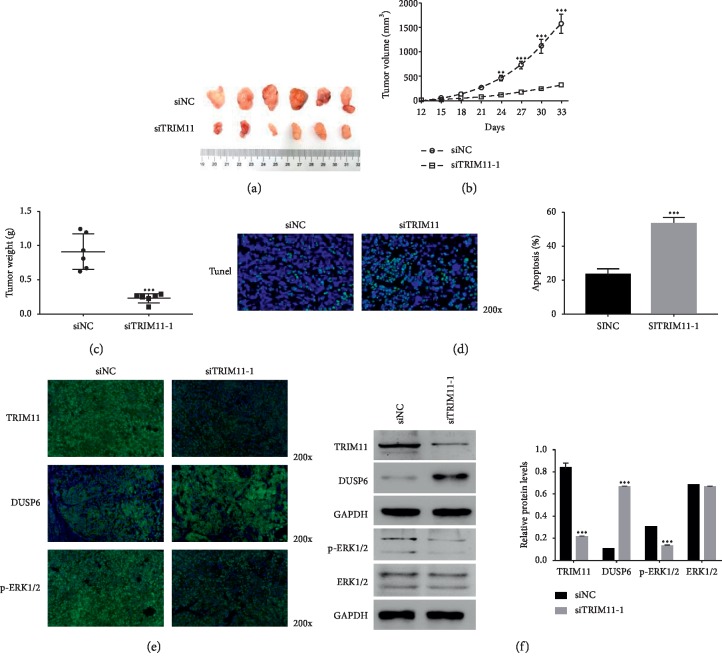
Knockdown of TRIM11 decreased the tumorigenicity of OS cells *in vivo.* (a) Tumors were removed from nude mice that were injected with siNC or siTRIM11-1 transfected cells. (b, c) Knockdown of TRIM11 significantly suppressed tumor growth *in vivo.*^*∗∗∗*^*p* < 0.001 vs siNC. (d) Cell apoptosis was upregulated in siTRIM11-1 tumors than in siNC tumors. ^*∗∗∗*^*p* < 0.001 vs siNC. (e) Immunofluorescence detection assay was used to examine the level of TRIM11, DUSP6, and ERK1/2 in siNC and siTRIM11-1 tumors, respectively. Magnification, 200x. (f) Western blot was used to examine the protein content of TRIM11, DUSP6, p-ERK1/2, and ERK1/2 in siNC or siTRIM11-1 tumors. ^*∗∗∗*^*p* < 0.001 vs siNC.

Next, we quantified TRIM11 function *in vivo* via constructing the OS model on nude mice. HOS cells that were transfected with siNC or siTRIM11 were subcutaneously inoculated into the armpit of nude mice (*n* = 6). Obviously, both siNC and siTRIM11 transfected cells were able to form tumors in nude mice (Figure 5(a)). However, the tumor volume and weight of siTRIM11 tumors were much lower than those of siNC tumors (Figures 5(b) and 5(c)). Moreover, the TUNEL staining assay indicated that the apoptosis rate of siTRIM11 tumors was significantly upregulated compared with that of siNC tumors (Figure 5(d)). Overall, these results demonstrated that TRIM11 silencing significantly reduced the tumorigenicity of OS cells *in vivo.*

Furthermore, the immunofluorescence assay was performed to quantify the protein contents of TRIM11, DUSP6, and p-ERK1/2 siNC and siTRIM11 tumors. As shown in Figure 5(e), the level of TRIM11 was much lower in siTRIM11 tumors than in siNC tumors. Importantly, TRIM11 silencing significantly promoted the contents of DUSP6 in xenografts. More importantly, the phosphorylation of ERK1/2 was downregulated in siTRIM11 tumors. Furthermore, the results obtained from western blot presented the similar results (Figure 5(f)). Taken together, these findings demonstrated that TRIM11 was a critical regulator in DUSP6 and ERK1/2 pathways *in vivo*.

## 3. Discussion

OS is one of the malignant tumors that deeply threaten human life quality. Although the adjuvant chemotherapy after tumor resection of OS has upregulated 10-year overall survival from 30% to 50% of patients in the 1970s, there is no significant increase since the 1990s [20]. Thus, gaining a deep insight into the molecule mechanism of OS is a critical step for developing novel therapy in the treatment for OS. In the present research, we identified that TRIM11 was an oncogene in the progression of OS. Moreover, our findings illustrated the potential role of TRIM11 as a target in the treatment of OS.

It has been demonstrated that TRIM11 upregulation contributes to the growth and metastasis of hepatocellular cancer cells as well as ovarian cancer cells [21, 22]. In the present analysis, TRIM11 silencing deeply suppressed the proliferation and improved the apoptosis of OS cells. Meanwhile, the similar results were also obtained in TRIM11 overexpression cells. These results firstly demonstrated that TRIM11 was a pro-proliferation and antiapoptosis factor in OS cells. TRIM11 overexpression accelerated the growth of OS cells.

Growing evidence has demonstrated that the activation of the ERK1/2 pathway accelerated the proliferation of tumor cells, including oral cancer [23], nasopharyngeal cancer [24], and hepatoma cells [25]. In this analysis, our findings revealed that overexpression of TRIM11 promoted the activation of the ERK1/2 signaling pathway in OS cells. Moreover, the ERK1/2 inhibitor PD98059 deeply blocked the function of TRIM11 in the growth of OS cells. Therefore, TRIM11 might promote the growth of OS cells through activating the ERK1/2 signaling pathway in OS cells.

Moreover, we found TRIM11 overexpression did not affect DUSP6 transcription but suppressed its translation in OS cells. Further analyses indicated that TRIM11 interacted with DUSP6 and promoted its ubiquitination in OS cells. Thus, TRIM11 overexpression might inhibit the translation of DUSP6 via enhancing its ubiquitination in OS cells.

Furthermore, previous reports have demonstrated that DUSP6 acts as an ERK1/2-specific dual-specificity phosphatase and functioned as a negative regulator of the ERK1/2 signaling pathway [26, 27]. These results revealed TRIM11 might be a novel component in the DUSP6/ERK1/2 signaling pathway in OS cells. TRIM11 might improve the phosphorylation of ERK/1/2 via inhibiting the translation of DUSP6 in OS cells. Much importantly, TRIM11 silencing deeply reduced the tumorigenicity of OS cells *in vivo.* Therefore, TRIM11 presented the potential value as a target in the treatment of OS.

## 4. Conclusion

In brief, we analyzed the function of TRIM11 in OS cells. Our results demonstrated that TRIM11 was a pro-proliferation and antiapoptosis factor in OS cells. Moreover, TRIM11 interacted with DUSP6 and involved in the ERK1/2 signaling pathway in OS cells. Our findings not only elucidated that TRIM11 was an oncogene gene in the progression of OS but also demonstrated its potential signaling pathway in OS cells.

## 5. Material and Methods

### 5.1. Cell Culture

All the cells involved in this study were purchased from the cell bank of Shanghai Biology Institute (Shanghai, China), including 143B, HOS, MG63, SAOS2, U2OS, and hFOB1.19. Cells were seeded in the DMEM medium (Trueline, USA) containing FBS (10%, 16000-044, GIBCO, USA) and penicillin-streptomycin solution (1%, P1400-100, Solarbio, China). All cells were cultured in the incubator with the condition of 5% CO_2_ at 37°C. The ERK1/2 inhibitor PD98059 (10 *μ*mol/L; S1177, Selleck, USA) was dissolved in DMSO (D2650, Sigma, USA) and used to culture cells.

### 5.2. RNA Extraction and Real-Time PCR

Total RNA was extracted using the TRIzol Reagent kit (1596-026, Invitrogen, USA) and converted to cDNA using the cDNA synthesis kit (#K1622, Fermentas, Canada). Experiment was established on a Real-time Detection (ABI-7300, ABI, USA) using an SYBR Green master mix (#K0223, Thermo, USA). The conditions of thermocycling were set as follows: 95°C for 10 minutes followed by 40 cycles of 95°C for 15 s and 60°C for 45 s. Relative gene expression determination was counted according to the 2^−ΔΔCt^ method using GAPDH as the endogenous reference. Three replications were necessary for all reactions. Supplementary [Supplementary-material supplementary-material-1] lists the primer sequences used in this study.

### 5.3. Silencing and Overexpression of TRIM11 in OS Cells

For the silencing human gene TRIM11 (NM_145214.2), three short interference RNAs that target different regions of TRIM11 were synthesized (Major, Shanghai, China) and subsequently transfected into U2OS and HOS cells, respectively, by using Lipofectamine 2000 (Invitrogen, USA). Meanwhile, a nonspecific scrambled siRNA was used as negative control (siNC). The targeting locus and sequence of siTRIM11 are provided in Supplementary [Supplementary-material supplementary-material-1].

As for the overexpression of TRIM11, the full length of TRIM11 cDNA was inserted into the lentiviral plasmid (pLVX-puro). Then, the recombinant vector (oeTRIM11) and a mock plasmid (oeNC) were transiently transfected into SAOS2 cells. Analyses were started at 48 h after transfection.

### 5.4. Western Blot

Total protein was extracted using the RIPA lysis buffer (JRDUN, Shanghai, China). The BCA protein assay kit (Thermo Fisher, USA) was utilized to measure total protein. 25 *μ*g protein of each sample was fractionated via running on SDS-PAGE (10%) and subsequently transferred onto the PVDF nitrocellulose membrane (HATF00010, Millipore, USA) for 12 h. Then, the membranes were probed with the primary antibodies at 4°C overnight followed by the appropriate HRP-conjugated goat anti-rabbit IgG (A0208, Beyotime, China). Protein signals were analyzed using a chemiluminescence system. GAPDH served as an endogenous reference. Each analysis was established in triplicate. Supplementary [Supplementary-material supplementary-material-1] provides the details of primary antibodies.

### 5.5. Cell Proliferation

Cell proliferation was determined by using Cell Counting Kit-8 (CCK-8) assay kits (CP002, SAB, USA) in accordance with the manufacturer's instructions In brief, the cells transfected as indicated were planted in 96-well plates and cultured for 0, 12, 24, and 48. OD 450 nm values of different cells were measured via using a microplate reader (DNM-9602, Pulangxin, China). Independent experiments in triplicate were needed for each time point.

### 5.6. Cell Apoptosis

In brief, cell apoptosis was examined by using the Annexin V-fluorescein isothiocyanate (FITC) apoptosis detection kit (C1063, Beyotime, China). All procedures were performed in accordance with the manufacturer's instructions. A flow cytometer (Accuri C6, BD, USA) was used to determine cells at 48 h after infection. Three replications were needed for each sample.

### 5.7. Co-Immunoprecipitation (Co-IP)

In brief, whole-cell extracts were isolated after transfection or stimulation with appropriate ligands. Then, all samples were incubated by the appropriate antibodies plus Protein A/G beads (Santa Cruz Biotechnology, USA) overnight. Beads were washed five times and separated by SDS-PAGE. Western blot was performed as indicated above.

### 5.8. Ubiquitination Assay

SAOS2 cells that were transfected with oeNC or oeTRIM11 were lysed by sonication in the 1% SDS-containing radio immunoprecipitation assay (RIPA) buffer on ice. Then, lysates were treated by Protein A/G PLUS-Agarose (sc-2003, Santa Cruz Biotechnology, USA) for 1 h. After that, each sample was incubated with the IgG (sc-2027, Santa Cruz Biotechnology, USA) overnight at 4°C. Then, the nuclear pellet was gathered by centrifugation at 3000 rpm for 5 min at 4°C and subsequently washed four times by Protein A/G Plus-Agarose beads. The purified proteins were run on 4–20% gradient SDS-PAGE. Anti-DUSP6 antibody (ab76310, Abcam, UK) and anti-ubiquitin antibody (ab7780, Abcam, UK) were used for immunoblotting.

### 5.9. Animal Experiment

This section was performed on the basis of the institute's guidelines for animal experiments and was recognized by the independent ethics committee of Zhejiang Rongjun Hospital, Zhejiang, China. All the experiments were conducted in accordance with the Institutional Animal Care and Use Committee (IACUC). A total of 5 × 10^6^ HOS cells that were transfected with siNC or siTRIM11-1 were subcutaneously injected into nude mice (*n* = 6 for each group; 4–6 week old, Shanghai Laboratory Animal Company, China). 12 days after injection, tumor length and width were measured every 3 days for 33 days. Tumor volume was determined according to length × (width^2^/2). All mice were sacrificed via cervical dislocation at day 42 after injection, and tumor tissues were removed from the xenograft mice and fixed in 4% formalin for further analysis.

### 5.10. TdT-Mediated DUTP Nick End Labeling (TUNEL) Staining Assay

The TUNEL assay kit (11684817910, Roche, Germany) was used to analyze the cell apoptosis of siNC and siTRIM11-1 tumor sections. All the procedures were performed in accordance with the instruction of the manufacturer. TUNEL-positive nuclei were counted in 3 different regions of each group.

### 5.11. Immunofluorescence Detection

In brief, the tissue sections were deparaffinized in xylenes and rehydrated through graded (100%–95%–70%) ethanols to distilled water. Then, sodium citrate buffer (0.01 M) was used for antigen retrieved with high pressure conditions for 15 min. After that, samples were washed with phosphate-buffered saline (PBS; 0.02 M) for 3 minutes three times at room temperature. Subsequently, samples were incubated with the rabbit anti-TRIM11 (10851-1-AP, Proteintech, USA), anti-DUSP6 (ab220811, Abcam, UK), and anti-ERK1/2 (ab184699, Abcam, UK) antibodies in PBS overnight at 4 °C followed by Alexa Fluor 488 goat anti-rabbit IgG (H + L) (A0423, Beyotime, China) for 1 hour at room temperature. Images were acquired by an ECLIPSE Ni microscope and a digital image analyzer (NIKON, Japan). Three replicates were needed for each analysis.

### 5.12. Statistical Analysis

All data were represented as mean ± SEM from three independent experiments and analyzed with the GraphPad Prism software, version 7.0 (CA, USA). The result was assessed by analysis of variance (ANOVA). *P* < 0.05 was accepted as statistical significance.

## Data Availability

The data used to support the findings of this study are included within the article.

## Supplementary Materials

Supplementary Materials Supplementary File 1: details of primer sequence. Supplementary Table 1: homosapiens tripartite motif containing 11 (TRIM11) (NM_145214.2) RNAi targeting locus information. Supplementary Table 2: details of the primary antibodies.
