# Supplementary material for: Tripartite Motif Containing 11 Interacts with DUSP6 to Promote the Growth of Human Osteosarcoma Cells through Regulating ERK1/2 Pathway
Source: Biomed Res Int. 2019 Dec 25;2019:9612125. doi: 10.1155/2019/9612125 (PMC6948331; doi:10.1155/2019/9612125)
Supplement: Supplementary Materials — Supplementary File 1: details of primer sequence. Supplementary Table 1: homosapiens tripartite motif containing 11 (TRIM11) (NM_145214.2) RNAi targeting locus information. Supplementary Table 2: details of the primary antibodies. [file 9612125.f1.pdf]

Supplementary File1: Primer sequence information

1.1 Homo sapiens tripartite motif containing 11 (TRIM11), mRNA

NM_145214.2

Primer F 5' CACCTAAGCTGCACAGTTCC 3'

Primer R 5' GGCTGCCTCCTAATTCTTCC 3'

Pos: 2008- 2192

Amplified product: Size: 185 bps

1.2 Homo sapiens dual specificity phosphatase 6 (DUSP6), transcript variant 1, mRNA

NM_001946.4

Primer F 5' CGATGAACGATGCCTATGAC 3'

Primer R 5' CTGATGCTGCCAAGAGAAAC 3'

Pos: 1406- 1674

Amplified product: Size: 269 bps

1.3 Homo sapiens glyceraldehyde-3-phosphate dehydrogenase (GAPDH), transcript variant 2, mRNA

NM_001256799.2

Primer F 5' AATCCCATCACCATCTTC 3'

Primer R 5' AGGCTGTTGTCATACTTC 3'

Pos: 436-653

Amplified product: Size: 218 bps

Supplementary Table1: Homo sapiens tripartite motif containing 11 (TRIM11) (NM_145214.2) RNAi targeting locus information

RNAi Targeting Locus		Sequence
Name	locus positon	
siTRIM11-1	672-690	GGAGAAGTCACTGGAGCAT
siTRIM11-2	708-726	GGATGCGTTGCTGTTCCAA
siTRIM11-3	746-764	GCGTCTTGTGGCAGAAGAT

Supplementary Table 2: The primary antibodies information

Antibody name	Catalog	Source	Dilution factor
TRIM11	Ab111694	Abcam, UK	1:2000
DUSP6	Ab238512	Abcam, UK	1:500
Bcl2	Sc-492	Santa，USA	1:300
c-fos	Ab184666	Abcam, UK	1:1000
ERK1/2	#9102	CST, USA	1:1000
p-ERK1/2	#9101	CST, USA	1:1000
GAPDH	#5174	CST, USA	1/2000
